# Sampling environmental DNA from trees and soil to detect cryptic arboreal mammals

**DOI:** 10.1038/s41598-023-27512-8

**Published:** 2023-01-05

**Authors:** Michael C. Allen, Robert Kwait, Anthony Vastano, Alex Kisurin, Isabelle Zoccolo, Benjamin D. Jaffe, Jordan C. Angle, Brooke Maslo, Julie L. Lockwood

**Affiliations:** 1 Ecology, Evolution and Natural Resources, Rutgers University, 14 College Farm Road, New Brunswick, NJ 08901 USA; 2 ExxonMobil Biomedical Sciences Inc, Annandale, NJ USA; 3 ExxonMobil Upstream Research Company, Spring, TX USA

**Keywords:** Bioinformatics, Ecology, Biodiversity, Community ecology, Conservation biology

## Abstract

Environmental DNA (eDNA) approaches to monitoring biodiversity in terrestrial environments have largely focused on sampling water bodies, potentially limiting the geographic and taxonomic scope of eDNA investigations. We assessed the performance of two strictly terrestrial eDNA sampling approaches to detect arboreal mammals, a guild with many threatened and poorly studied taxa worldwide, within two central New Jersey (USA) woodlands. We evaluated species detected with metabarcoding using two eDNA collection methods (tree bark vs. soil sampling), and compared the performance of two detection methods (qPCR vs. metabarcoding) within a single species. Our survey, which included 94 sampling events at 21 trees, detected 16 species of mammals, representing over 60% of the diversity expected in the area. More DNA was found for the 8 arboreal versus 8 non-arboreal species detected (mean: 2466 vs. 289 reads/sample). Soil samples revealed a generally similar composition, but a lower diversity, of mammal species. Detection rates for big brown bat were 3.4 × higher for qPCR over metabarcoding, illustrating the enhanced sensitivity of single-species approaches. Our results suggest that sampling eDNA from on and around trees could serve as a useful new monitoring tool for cryptic arboreal mammal communities globally.

## Introduction

A lack of knowledge of species distributions and population trends impedes conservation efforts for terrestrial mammals, a quarter of which are at risk of extinction^1–3^. Cryptic arboreal mammal species, including tree-roosting bats, are especially lacking in data as a result of labor-intensive survey methods coupled with inadequate resources for detailed investigations^4–6^. Because robust and long-term monitoring is vital to conservation, developing and improving tools that can more efficiently track population changes and assess species distributions is critically important^2,7^. Here we contribute to these efforts by trialing novel and established environmental DNA (eDNA) sampling methods on and around individual trees to detect arboreal mammals.

While a variety of detection methods exist for monitoring cryptic arboreal mammals, most tend to be labor intensive, suffer from poor detection rates^4,8,9^, and can be biased towards more easily observed species^10^. For example, among small bats (Microchiroptera), the demographic datasets available for species that roost in easily accessible locations, such as human structures, are substantially more robust than those for species roosting in inaccessible locations such as tree canopies and rock crevices^6^. The combination of capture, visual search, and telemetry methods necessary to locate bats in tree canopies and rock crevices is labor intensive, requiring hours of diurnal and nocturnal fieldwork to catch and then follow individual animals to roosts^11^. Visual transect surveys are another common, fieldwork-intensive survey method for canopy-dwelling mammals that, when targeting nocturnal species, must be conducted at night via spotlight^4,8,9^. In the past two decades, advances in technology and an increasingly acute need for monitoring data have spurred a flurry of research into novel methods to enhance detection of these cryptic arboreal species, including bioacoustics^12,13^, drone- or canopy-mounted cameras^8,14^, scent detection dogs^15^, and eDNA-based methods^16–18^. The ability to survey for arboreal mammals based on shed DNA may have distinct advantages over conventional methods that require separate surveys for diurnal and nocturnal species (e.g., visual methods), or have difficulty discriminating among groups of species (e.g., acoustic methods^19^).

Protocols for detecting the presence of aquatic or semi-aquatic vertebrates, including mammals, based upon shed DNA are now in widespread use^20,21^. For fully terrestrial species, eDNA detection techniques represent a rapidly expanding area of research^22–24^. Building off of early efforts at recovering DNA from physical traces such as tracks, hair, and scat^25,26^, mammal researchers have recently branched out into sampling communities using eDNA that has been deposited or transported into ponds and rivers^16,27–30^ or left on attractants such as natural saltlicks or hollow logs^31,32^, including for some cryptic arboreal species^18^. While such approaches are appealing for their ability to produce community-level inventories at large spatial scales, sampling is limited to wherever such natural features occur and is therefore inadequate for finer-scale monitoring. For example, such methods cannot be used to survey for cryptic arboreal species at the level of individual forested sites or trees if no water bodies or natural attractants are present nearby. Such finer scale resolution may be necessary to trigger regulatory protection or management interventions within jurisdictional boundaries (e.g., sites of proposed development, multi-use public parcels).

Fully terrestrial eDNA-based techniques to sample vertebrates (i.e., those not tied to water bodies) have included sampling eDNA found in soil or air^17,23,32^, and testing residues left on artificial attractants such as coverboards^22,24^. Such methods circumvent the geographic limitations of those tied to water by allowing researchers to decide precisely where in the landscape to take samples. These approaches may be particularly promising in the context of sampling for cryptic arboreal mammals as they allow targeted sampling near individual trees or other sites with unknown occupancy status. For example, soil and air samples taken from caves and enclosures were demonstrated to readily allow detection of eDNA from big brown bats (*Eptesicus fuscus*)^17^. Leempoel et al.^23^ demonstrated that soil eDNA samples collected at camera trapping stations performed as well as cameras for larger mammals, and better than cameras for smaller species. Extending these terrestrial eDNA-based techniques to sample for cryptic arboreal species could involve either testing soil beneath individual trees or using surface sampling techniques to recover eDNA from bark. A newly developed tool for sampling surface eDNA using damp commercially available paint rollers performed exceptionally well for recovering arthropod DNA from tree bark^33^ and reptile DNA from cover objects^24^, and for identifying the vertebrate occupants of tree holes^18^. However, it has yet to be extended to sampling the surface of tree trunks to detect arboreal mammals despite its obvious promise.

Judging the performance of eDNA survey methods requires quantitative evaluation and comparison of their effectiveness and efficiency relative to conventional methods^8,23,30^. We evaluated the mammal communities detected by two eDNA recovery methods—soil sampling around trees and roller sampling of tree bark—in two New Jersey, USA woodlands. Our primary objectives were to: (1) evaluate if eDNA metabarcoding of bark and soil samples can effectively characterize arboreal mammal communities, including tree-roosting bats, a group that includes several cryptic and threatened species in our region; (2) quantitatively compare the metabarcoding detection rates of mammals between bark and soil eDNA samples; and (3) evaluate the extent to which a qPCR molecular detection approach can improve detection rates over metabarcoding for a single species, the big brown bat.

## Methods

Our methods consisted of collecting soil and roller eDNA samples at 21 focal trees at two sites; performing eDNA metabarcoding on these samples with a mammal-focused primer set; using a community occupancy model to compare detection rates among species and between collection methods; and assessing the relative ability of these methods to characterize the community of likely arboreal and non-arboreal mammals present. We further compared the ability of a targeted qPCR assay to detect eDNA of one species (big brown bat) in samples relative to metabarcoding.

### Study area and target species

Field sampling occurred within the Rutgers Ecological Preserve (40.52° N, − 74.44° W; ~ 130 ha) and Morristown National Historical Park (40.77° N, − 74.54° W; ~ 590 ha) in New Jersey, USA. Both sites are dominated by mixed-age second growth Oak-Hickory forest, which is common in the region^34^. We built a database of mammal species occurring at the sites from personal records (unpublished data), park websites, the National Park Service’s Inventory and Monitoring Program database, a camera trapping study^35^, and iNaturalist^36^ (see Table 1). This database formed the basis for reference for any species detected.

**Table 1:** All mammal species detected using eDNA metabarcoding or identified as likely to be present based on other sources^a^ at Rutgers Ecological Preserve and Morristown National Historic Park in New Jersey, USA.

Clade	Species	Arboreal^b^	Rutgers Ecological Preserve		Morristown National Historic Park	
			Previously documented (references)^a^	Detected with metabarcoding (% of samples)^c^	Previously documented (references)^a^	Detected with metabarcoding (% of samples)^c^
Bats (Chiroptera)^d^	*Eptesicus fuscus* (Big brown bat)	Yes	Yes (A, B)	Yes (R: 7%)	Yes (A, B)	Yes (R: 3%)
	*Lasiurus borealis* (E. red bat)	Yes	Yes (A, B)	No	Yes (A, B)	No
	*Myotis septentrionalis* (N. long-eared bat)	Yes	Yes (A, B)	No	Yes (A)	No
	*Myotis leibii* (E. small-footed bat)	No	No	No	Yes (B)	Yes (R: 3%, S: 5%)
	*Lasionycteris noctivagans* (Silver-haired bat)	Yes	Yes (A, B)	No	Yes (B)	No
	*Lasiurus cinereus* (Hoary bat)	Yes	No	No	Yes (B)	No
Rodents (Rodentia)^d^	*Sciurus carolinensis* (E. gray squirrel)	Yes	Yes (C, D)	Yes (R: 78%, S: 44%)	Yes (A, C, E)	Yes (R: 52%, S: 43%)
	*Glaucomys volans* (S. flying squirrel)	Yes	Yes (A, B)	Yes (R: 52%, S: 19%)	Yes (B)	Yes (R: 47%, S: 26%)
	*Tamiasciurus hudsonicus* (American red squirrel)	Yes	No	No	Yes (A, C, E)	Yes (R: 6%)
	*Tamias striatus* (E. chipmunk)	Yes	Yes (C)	Yes (R: 56%, S: 38%)	Yes (C, E)	Yes (R: 25%, S: 16%)
	*Marmota monax* (Groundhog)	No	Yes (A, C)	No	Yes (C, E)	No
	*Microtus sp.* (Vole sp.)	No	No	Yes (S: 6%)	No	No
	*Peromyscus leucopus* (White-footed mouse)	Yes	Yes (D)	Yes (R: 19%)	Yes (E)	Yes (R: 13%)
	*Rattus norvegicus* (Brown rat)	No	No	Yes (R: 4%)	No	No
Rabbits (Lagomorpha)	*Sylvilagus floridanus* (E. cottontail)	No	Yes (A, C)	Yes (R: 4%)	Yes (A, C, E)	No
Insectivores (Eulipotyphla)	*Blarina brevicauda* (N. short-tailed shrew)	No	Yes (C)	No	Yes (C, E)	No
	*Scalopus aquaticus* (E. mole)	No	Yes (C)	No	Yes (C)	No
Carnivores (Carnivora)^d^	*Procyon lotor* (Raccoon)	Yes	Yes (C, D)	Yes (R: 41%)	Yes (A, E)	Yes (R: 9%, S: 16%)
	*Mephitis mephitis* (Striped skunk)	No	Yes (D)	No	Yes (A, E)	No
	*Vulpes vulpes* (Red fox)	No	Yes (C, D)	Yes (R: 15%)	Yes (A)	Yes (R: 3%)
	*Canis latrans* (Coyote)	No	Yes (D)	No	Yes (A, E)	No
	*Canis lupus familiaris* (Domestic dog)	No	No	Yes (R: 19%)	No	Yes (R: 28%)
	*Felis catus* (Domestic cat)	No	Yes (C, D)	Yes (R: 4%)	No	Yes (R: 3%)
Opossum (Didelphimorphia)	*Didelphis virginiana* (Virginia opossum)	Yes	Yes (D)	Yes (R: 4%)	Yes (E)	Yes (R: 6%, S: 5%)
Deer (Artiodactyla)	*Odocoileus virginianus* (White-tailed deer)	No	Yes (A, C, D)	Yes (R: 52%, S: 19%)	Yes (A, C, E)	Yes (R: 28%, S: 11%)

^a^References for “Previously documented” status: A—Websites for Rutgers Ecological Preserve (https://ecopreserve.rutgers.edu/; accessed 6/10/2022) and Morristown National Historic Park (https://www.nps.gov/morr/learn/nature/mammals.htm; accessed 6/10/2022); B—our own field observations during a 2-year field study; C—iNaturalist ‘research grade’ observations^36^; D—a 2019 camera trapping study at Rutgers Ecological Preserve^35^; E—National Park Service Inventory and Monitoring Program mammal checklist for Morristown National Historic Park (https://irma.nps.gov/NPSpecies/Search/SpeciesList/MORR; accessed 6/10/2022).

^b^Defined as climbing or resting in trees as part of normal, daily foraging or roosting activities.

^c^R—detected in roller samples (Rutgers Ecological Preserve: n = 27 samples at 9 trees; Morristown National Historic Park: n = 32 samples at 12 trees); S—detected in soil samples (Rutgers Ecological Preserve: n = 16 samples at 6 trees; Morristown National Historic Park: n = 19 samples at 8 trees).

^d^Three additional species were identified as occurring at the sites but are omitted from this list because they were either filtered out of our metabarcoding results for contamination reasons (house mouse, *Mus musculus* [Rodentia]; little brown bat; *Myotis lucifugus* [Chiroptera]) or because the primer set is ineffective for related species (American black bear, *Ursus americanus* [Carnivora]).

### Field sampling

We cleaned and disinfected the sampling pole and soil collection spoons prior to each field use, and transported equipment in sterile bags to avoid contamination (Supplementary Table S1). To test for contamination of field gear, we collected a ‘field negative’ roller sample upon arriving at the field site (Morristown or Rutgers) by going through the same sample handling procedures described below but without field sampling.

Between July and September 2021, we sampled 21 trees: 12 at Morristown and 9 at Rutgers. Trees were selected on their suspected status as diurnal roosts from a parallel radio-telemetry tracking study involving three species: eastern red bat (*Lasiurus borealis*; n = 17 trees), northern long-eared bat (*Myotis septentrionalis*; n = 2 trees), and big brown bat (*Eptesicus fuscus*; n = 2 trees) (BM, unpublished data). Trees were deciduous (mainly *Liriodendron, Quercus, Fagus,* and *Betula*) and 17–81 cm in diameter (mean: 51 cm; see Supplementary Table S2). At 19 trees, we collected soil and roller eDNA within 24 h after a roosting bat was suspected present, and then again on the following two consecutive days (48 h, 72 h), for a total of three rounds of sampling per tree. Two trees received only the first round of sampling for logistical reasons. Thus, we collected a total of 59 sets of roller and soil samples at 21 trees.

We collected soil samples beneath the canopy of focal trees by establishing four transects, oriented 90 degrees from each other, and extending from the trunk out to ~ 3 m, near the outer edge of the canopy. The orientation of the four-way transects was rotated ~ 30 degrees during each subsequent sampling event to avoid overlapping previous sampling locations. Along each transect, we collected a ~ 20 g scoop of soil every 1 m (i.e., 4 per transect), and then combined the ~ 320 g of soil for each tree into a 178 × 305 mm sterile plastic bag. Samples were transported in an ice-filled cooler and stored at − 20 °C until processing.

We collected roller samples on each focal tree trunk following Valentin et al. including measures to avoid sample contamination (Supplementary Table S1). Applying the paint roller firmly to the bark of the tree, we rolled from the base of the trunk to a height of ~ 3 m around the entire circumference of the tree. Then, we removed the roller from the pole, sealed it in a 304 × 114 mm sterile plastic bag with ~ 400 mL of DI water, and shook the bag while massaging the roller for ~ 30 s to help move any eDNA into solution. We filtered the water using a self-desiccating filter assembly (Smith-Root, Inc., Vancouver, Washington, USA) equipped with a 10-μm polycarbonate track-etched (PCTE) filter membrane and a peristaltic pump. We then used sterilized forceps to remove the filter membrane from the assembly and place it into a 1.5 mL tube filled with 100% non-denatured ethanol. Tubes were stored at − 20 °C until extraction.

### DNA extraction and sequencing of environmental samples

For the 59 roller samples, we evaporated ethanol from filters using a vacuum centrifuge, extracted DNA using the DNeasy Blood and Tissue kit (Qiagen), and removed impurities using Ampure XP beads (Beckman Coultier, Pasadena, California, USA). For soil, we extracted DNA from a 5 mL portion of the sample using the DNeasy PowerMax Soil Kit (Qiagen) following manufacturer’s protocols. Unfortunately, we were unable to procure enough kits to extract all 59 soil samples due to prolonged shipping delays. To overcome this hurdle, we designed a subsampling scheme, based on haphazardly selecting trees from which to extract DNA from soil samples, until the soil extraction kits in our possession were exhausted. This resulted in a total of 35 soil samples analyzed from 14 trees (see Supplementary Table S2). We confirmed there was no sampling bias resulting from this subsampling scheme by performing a parallel analysis (see Statistical Analysis and Results). For both soil and roller samples, one extraction negative control was constructed for each extraction batch, consisting of PCR-grade water in place of the sample, but otherwise treated the same.

We prepared libraries using the Illumina two-step PCR metabarcoding protocol^37^. For each sample, we ran one initial PCR using the MiMammal-U primer set in replicates of 3 to reduce sample drop-out and PCR bias. The MiMammal-U primer set targets an ~ 200 bp region of the 12S locus and was created to specifically amplify mammal DNA from environmental samples^38^. We included at least one PCR negative control in each PCR run. Reaction preparation for the first PCR followed the Illumina protocol^37^ and had the following cycling parameters: 98 °C for 3 min, 35 cycles of 95 °C (30 s), 65 °C (30 s), and 72 °C (1 min), followed by final extension at 72 °C for 5 min, and then hold at 4 °C. For the soil samples, we used the same parameters except with an annealing temperature of 67 °C. The primers also contained partial Illumina adapter sequences to act as binding sites for a second PCR run (described below). We then pooled all the replicates for each sample. After pooling, we performed a bead clean-up for each sample to remove primer dimers and ran a second PCR to add the remainder of the Illumina adapter sequences and add sample-specific dual indexes. For the second PCR, reaction preparation and cycle parameters were identical to those in the Illumina protocol^37^. We performed agarose gel electrophoresis on each sample, and all libraries that produced a visible band were then cleaned with a second magnetic bead clean-up and quantified using the Qubit dsDNA High sensitivity DNA quantification assay. Libraries with no visible band on the gel were not analyzed further, with the exception of negatives, of which all were included. We then pooled all samples in equimolar ratios; 5 μl of each negative were also included in the final sequencing pool. We sent libraries to the Genomics Core Facility at Princeton University for sequencing on an Illumina MiSeq v3 2 × 300nt.

### Bioinformatics

We processed sequence data and made taxonomic assignments following OBITools^39^ documentation and Leempoel et al.^23^ (see Supplementary Table S3). Briefly, we aligned paired reads, and removed reads with join scores < 40, bases with quality scores < 30, and adapter sequences. We then removed reads with ≥ 21 ambiguous bases, dereplicated reads, removed sequences with read counts < 10 or with length < 80 bp, and filtered all reads for PCR or sequencing errors.

We generated a reference database from all vertebrate sequences available from ENSEMBL^40^ and used EcoPCR to match taxa to our sequence reads. For unmatched reads, we assigned taxa manually using BLAST^41^ and performed a local BLAST search against a supplemental database of 17 sequences from 9 bat species that we generated from tissue samples (see Supplementary Appendix S1 and Table S4). We then removed all resulting sequences with insert lengths ≥ 200 to exclude non-target taxa (e.g., bacteria)^23^. Additionally, we removed sequences from 10 species that were deemed to be likely contaminants from other projects in our lab (Supplementary Table S5) and five that are commonly associated with contamination in other metabarcoding studies^23,28,30^ (*Bos taurus*, *Homo sapiens*, *Mus musculus, Sus scrofa, Gallus gallus*). Following assignment and filtering of taxa, we controlled for contamination by setting a detection threshold for each OTU based on the number of reads that appeared in the negatives, removing any counts that appeared in field, extraction, and PCR negatives from the corresponding samples. Finally, we removed any OTU with a read count < 20 as an additional control for contamination and to reduce noise in the data set.

### Big brown bat qPCR methods

To compare quantitative PCR (qPCR) detection methods with metabarcoding, we re-tested the same 94 extracted roller and soil samples described above using a highly-sensitive qPCR assay for big brown bat that targets a 91 bp segment of mtDNA in the COI region (limit of detection: 2 copies per reaction)^17^. Our qPCR methods matched those of Serrao et al.^17^, except that we used a StepOne Plus Real-Time PCR System (Applied Biosystems, Inc., Waltham, Massachusetts, USA) and lacked an internal positive control. qPCR runs included 3 technical replicates each for all samples, a no-template control, and a six-level standard curve of synthetic DNA (10-fold dilution). Following Serrao et al.^17^, we confirmed species identification in all positive samples by enzymatically cleaning the resulting qPCR product (ExoSAP-IT™ PCR Product Cleanup Reagent, Applied Biosystems, Inc.) and performing bidirectional Sanger sequencing using the same primer set as for the qPCR reaction. The resulting sequences were aligned using Geneious Prime (Biomatters, Inc., Aukland, New Zealand) and identified to species using BLAST as described above.

### Statistical analysis

We assessed the performance of soil and roller eDNA sampling for metabarcoding analyses using a Dorazio-Royle (DR) community occupancy model^42^. These hierarchical models can estimate the effects of covariates on the probability of presence (occupancy) and detection given presence for multiple species simultaneously. Information is shared among species by treating species-specific covariate effects as originating from common distributions. In our model, the dependent variable was the detection (1) or non-detection (0) of mammal species at each focal tree during each sampling event. We included sampling method (soil or roller) as a covariate in the detection sub-model to evaluate differences in detection probability between the two methods*.* In addition, we visualized mammal community data using Venn diagrams, taxonomic heat trees^43^, species accumulation curves, and non-metric multidimensional scaling (NMDS) ordination plots^44^.

We compared the performance of qPCR versus metabarcoding methods for detecting big brown bat eDNA using multimethod occupancy models^45^. These multilevel models use joint detection information (e.g., from qPCR and metabarcoding) to inform the availability for detection, *θ*, and detection probability given availability, *P*(*detect* | *θ*)*.* In this case, availability refers to the probability of big brown bat eDNA presence within a sample. The dependent variable was the detection (1) or non-detection (0) of big brown bat by each method. We used a covariate for sampling method (soil vs. roller) within the availability sub-model, and a covariate for molecular approach (qPCR vs. metabarcoding) to estimate the method-specific probability of detecting big brown bat eDNA given availability within the sample. We then estimate the total ‘per-visit detection rate’ of bats at focal trees by multiplying *θ* by *P*(*detect* | *θ*). For both the DR and the multimethod occupancy model, we used the per-visit detection probability (*p*) to estimate the cumulative probability of detecting a species at least once given *n* repeated samples using the formula 1− (1 − *p*)^*n*^ (e.g., Ref.^24^).

All models were fit in a Bayesian framework with non-informative priors (see Ref.^42^, pp. 607 and 690) using JAGS and jagsUI in program R^46,47^. We ran 3 chains of 30,000 iterations each, including a burn-in period of 10,000, keeping every 10th draw. Model convergence was assessed by examining trace plots and Gelman-Rubin statistics (r-hat < 1.1). We compared estimates of per-visit detection probability for the various methods by examining and plotting posterior predictive distributions.

## Results

We performed roller and soil eDNA sampling at 21 trees, obtaining three samples per tree at all except for two trees, which each received a single sample. We analyzed all 59 roller samples from 21 trees, and a subset of 35 soil samples from 14 trees (1–3 samples per tree; mean = 2.5/tree). Forty-seven of 59 roller samples and 35 of 35 soil samples had measurable read counts with a total of 9,852,404 and 4,731,187 reads matched to roller and soil samples, respectively. After all filtering steps (see Supplementary Table S6), contaminant reads still remained in 5 of our 46 negative control samples, all associated with two taxa: *Canis lupus familiaris* (7702 reads of 4 OTUs) and *Glaucomys volans* (a single read of 1 OTU). After removal of these negative control reads from samples, 2,548,768 total reads across 46 samples remained for roller samples, and 33,314 total reads across 23 samples remained for soil samples (Supplementary Table S6).

**Figure 1:**
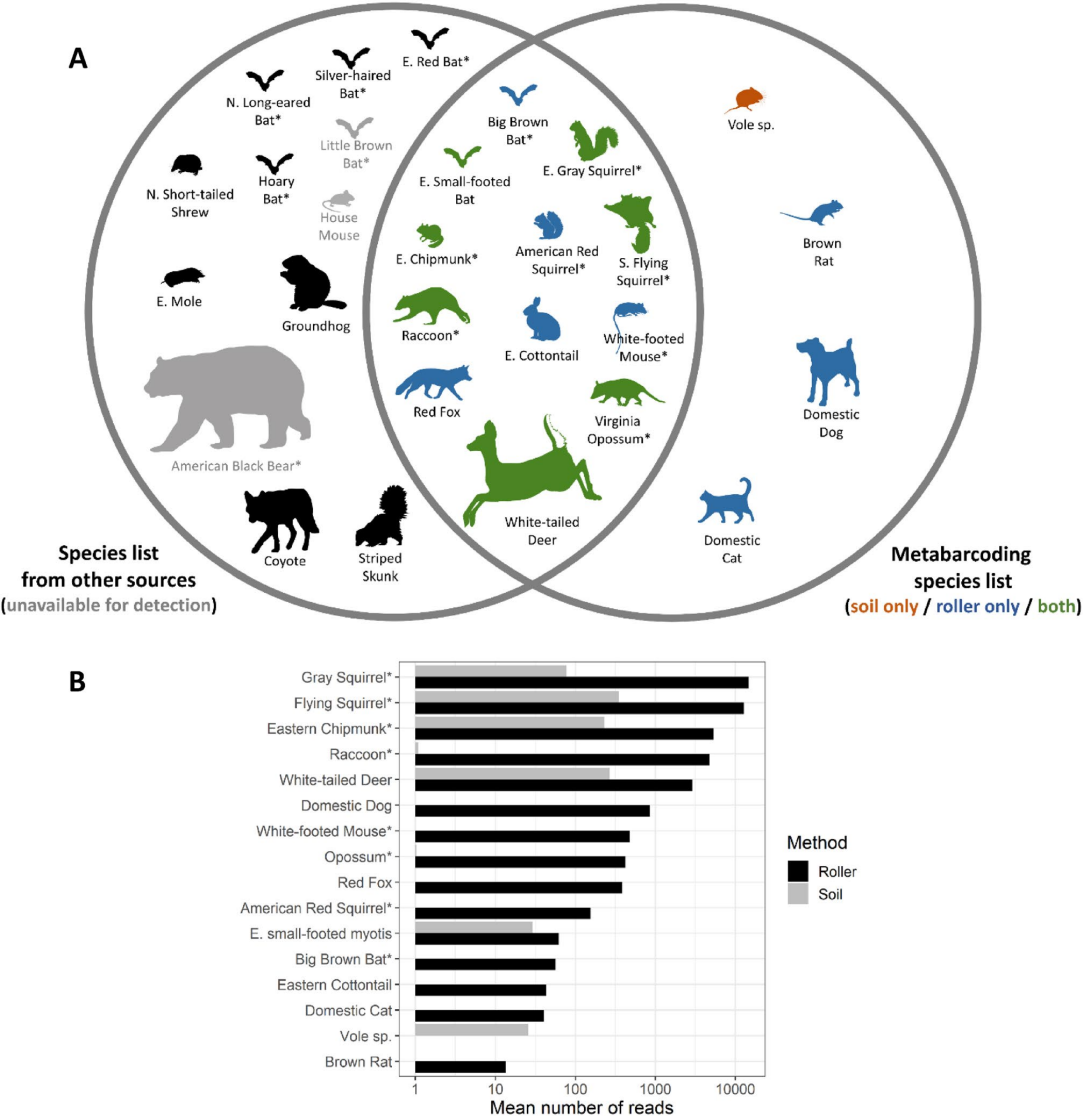
Mammal species detected with eDNA metabarcoding using soil and tree bark surface (‘roller’) sampling in two woodlands in central New Jersey, USA. Venn diagram (**A**) shows which species were detected with metabarcoding (right circle) and which were previously reported as present in the parks (left circle; see Table 1). Color coding: blue—detected with metabarcoding of roller samples only; orange—detected with metabarcoding of soil samples only; green—detected with metabarcoding of both roller and soil samples; black—not detected using metabarcoding; gray—species deemed unavailable for metabarcoding detection due to contamination (mouse and bat) or poor performance of the primer set (bear; see Methods). The bar graph (**B**) shows the mean number of metabarcoding reads per sample for each species detected. Arboreal species are identified with an asterisk (*). Images: www.phylopic.org (M. Michaud, D. Orr, T. Heath, F. Sayol, C. Schmidt, B. Barnes, A. Wilson, R. Groom, N. Skinner, G. Palomo Munoz, M. Keesey, S. McDavid).

**Figure 2:**
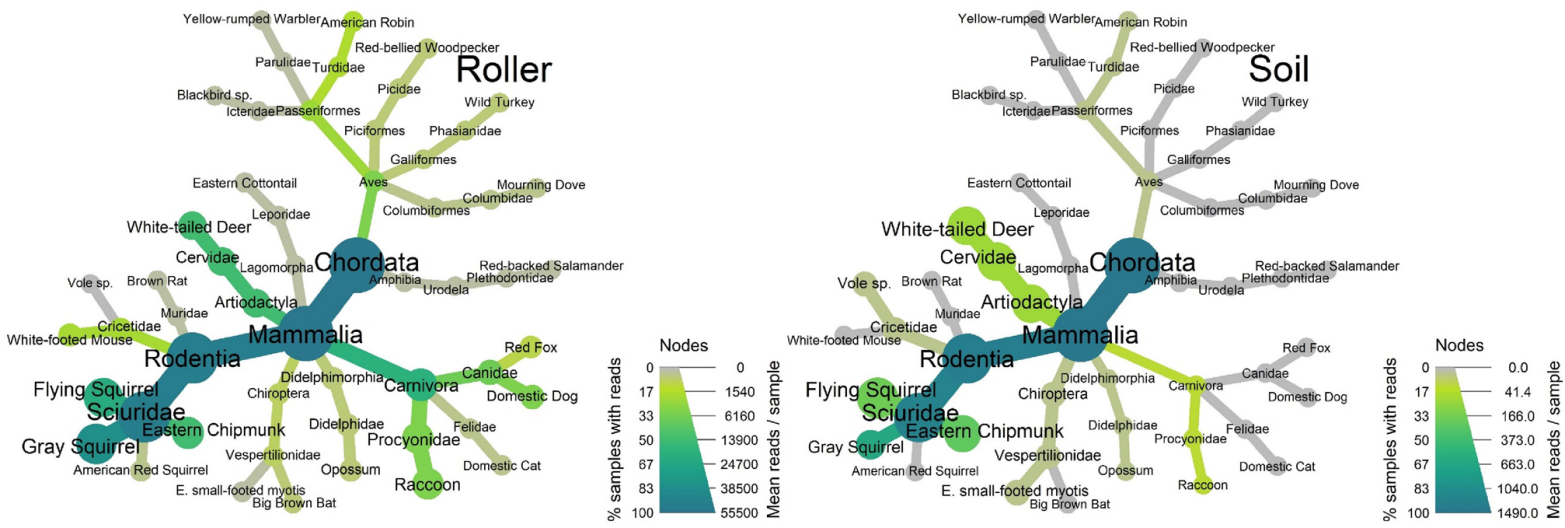
Taxonomic affiliation all species detected in a tree bark surface (‘roller’) and soil eDNA metabarcoding study in New Jersey, USA woodlands. The width of each node and branch shows the mean number of reads per sample, while the shading shows the percent of samples that included any reads.

A total of 359 OTUs remained after all filtering steps, attributable to 16 mammal species: 8 arboreal species (5 detected with soil, 8 with roller) and 8 non-arboreal species (3 detected with soil, 7 with roller; Figs. 1 and 2). We identified 28 total species (14 arboreal, 14 non-arboreal) as likely to be present at the sites based on our list compiled from external sources in addition to our metabarcoding results (Table 1, Fig. 1A). Two of those species (house mouse and little brown bat) were not available to be detected using metabarcoding methods as they were excluded due to contamination concerns (Table 1, Supplementary Table S5), and one species (American black bear) was not available for detection because the MiMammal-U primer set is not effective for bears^38^. Thus, the 16 species we detected using metabarcoding represents 64% of the 25 total species expected to be present and available for detection. Of the 9 species that were not detected using metabarcoding, 4 were considered arboreal, all of which were bats (Table 1, Fig. 1A). We detected 2 of 6 expected bat species: big brown bat, a tree-roosting species, and eastern small-footed bat, a species that roosts in rock crevices. Four species (brown rat, vole sp., domestic dog, and small-footed bat) were detected by metabarcoding but did not occur in our compiled list of likely species (Table 1, Fig. 1A).

In general, higher read counts were found in roller versus soil samples and for arboreal versus non-arboreal species. For roller samples, the mean number of reads per sample averaged 4849 ± 2094 (SE) across species for arboreal mammals (range = 56–14,699, n = 8), and 537 ± 353 for non-arboreal mammals (range = 0–2901, n = 8; Fig. 1B). For soil samples, the number of reads averaged 82 ± 48 for arboreal mammals (range = 0–349, n = 8), and 40 ± 33 (range = 0–270, n = 8) for non-arboreal mammals. The number of reads for bats averaged 59 ± 3 (range = 56–61, n = 2) for roller sampling and 14 ± 14 (range = 0–28, n = 2) for soil sampling. The discrepancy in mean read depth observed between sampling methods is partly explained by the removal of a large number of microbial reads from soil samples during bioinformatics (> 95% of reads removed for soil compared with ~ 50% of reads for roller; Supplemental Table S6).

**Figure 3:**
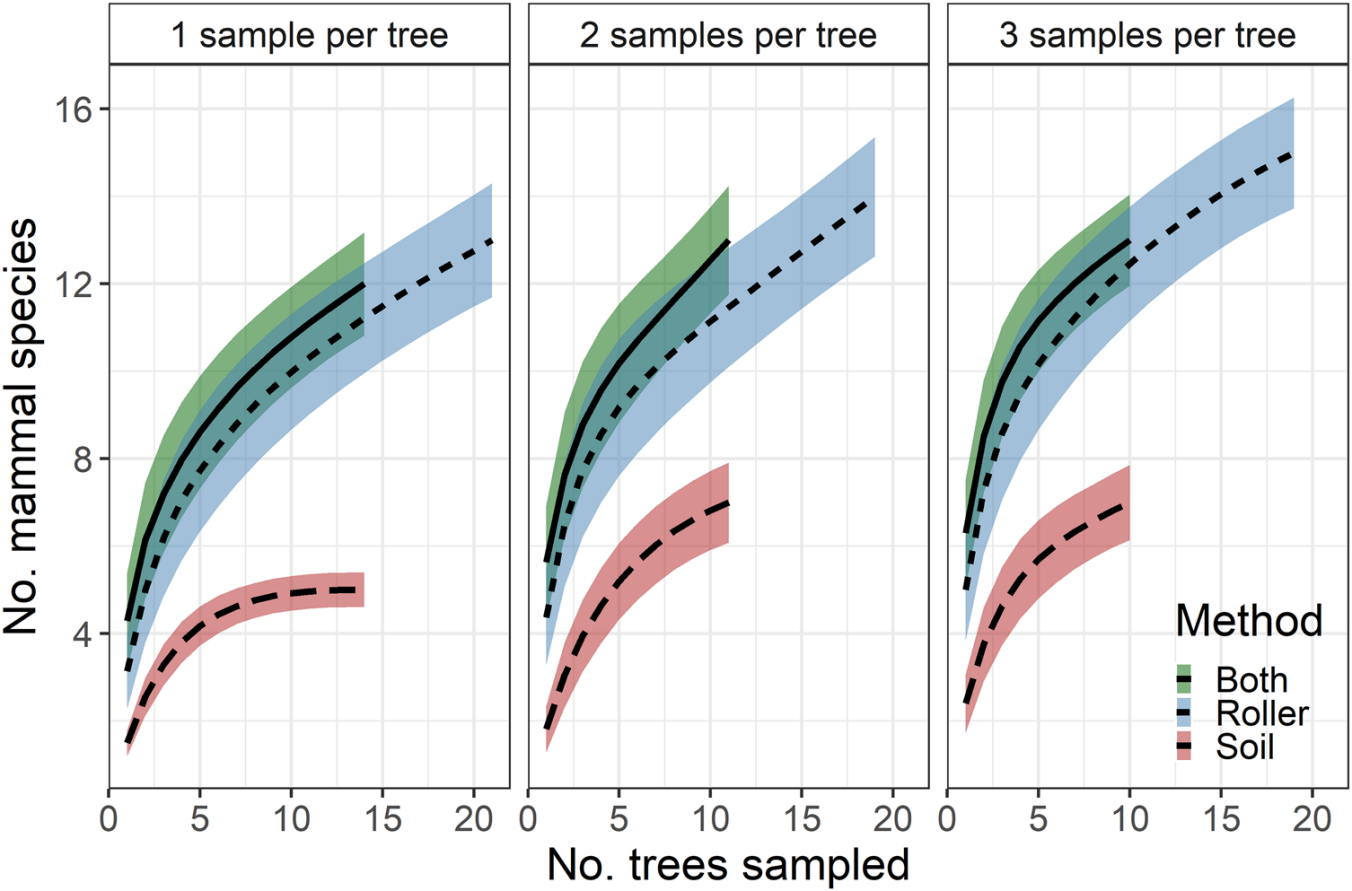
Species accumulation curves (mean ± 1 SD) for mammals detected using metabarcoding with tree bark surface (‘roller’) or soil eDNA sampling and varying numbers of sampling visits per tree (1–3). ‘Both’ refers to the pooled results at trees where both roller and soil sampling was performed.

Despite having fewer samples and fewer reads per sample, our soil method resulted in a generally similar characterization of mammal community composition to the roller method (Figs. 1 and 2; see also NMDS ordination plot, Supplementary Fig. S1). Species accumulation curves (Fig. 3) revealed that the number of species detected had not yet leveled off with either method, suggesting that sampling additional trees likely would have added more species. In addition to mammals, we also detected seven other vertebrates (six birds and one salamander), all of which were detected using roller methods, but only one of which (American Robin, *Turdus migratorius*) was detected using soil methods (Fig. 2).

**Figure 4:**
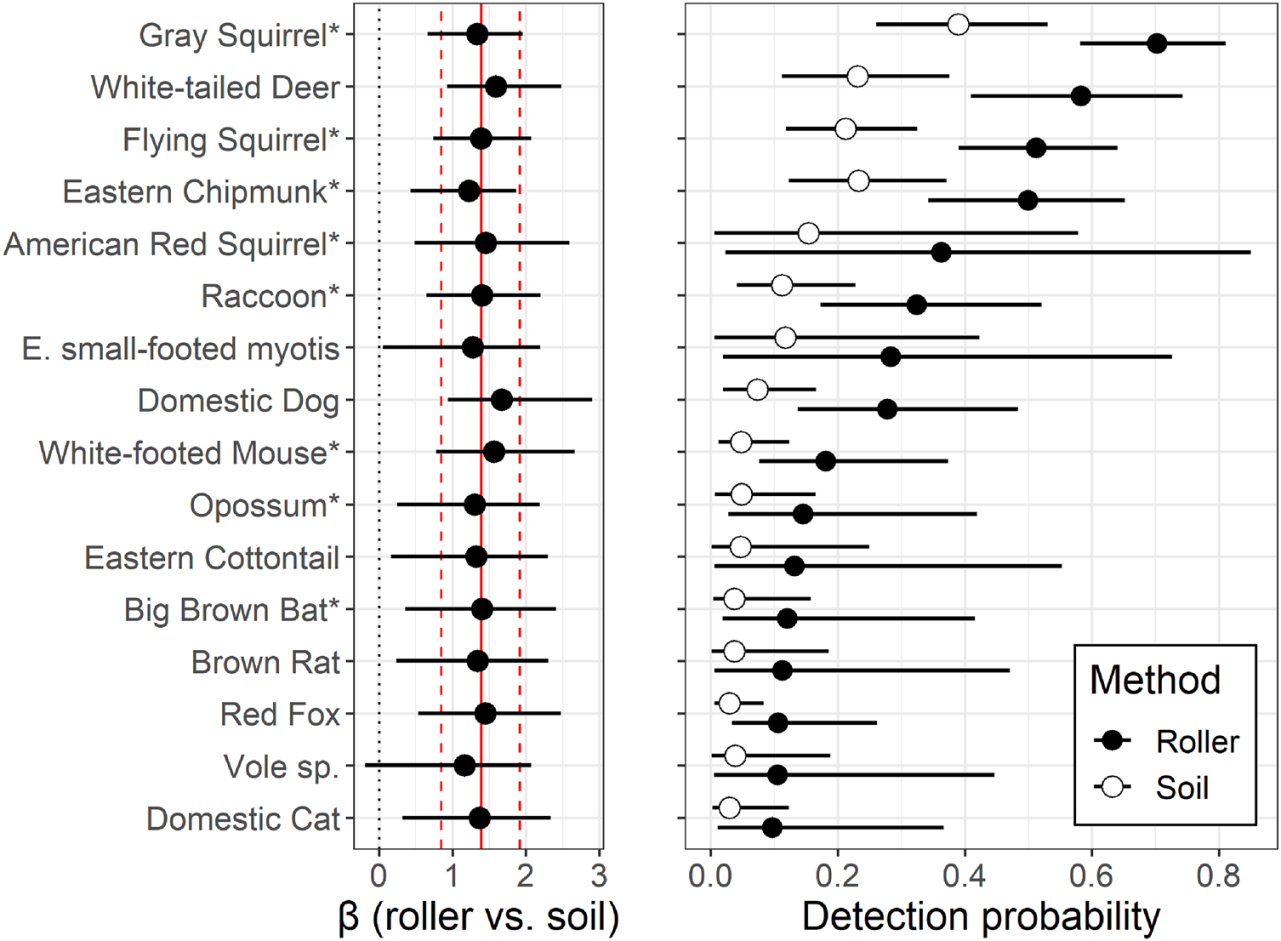
Results of a community occupancy model of mammal species detected in a tree bark surface (‘roller’) and soil eDNA metabarcoding study in New Jersey, USA woodlands. The left panel shows estimated slope coefficient for the effect of sampling method (roller vs. soil) on detection probability for each species (solid circles with 95% credible intervals) and for all species combined (solid vertical line with dashed lines indicating 95% credible intervals). A slope above zero (gray vertical dotted line) indicates higher detection probability for roller samples compared with soil samples. The right panel shows the model-estimated detection probability for each species with roller methods (solid circles) and soil (open circles) eDNA collection methods. Arboreal species are indicated with an asterisk (*).

**Figure 5:**
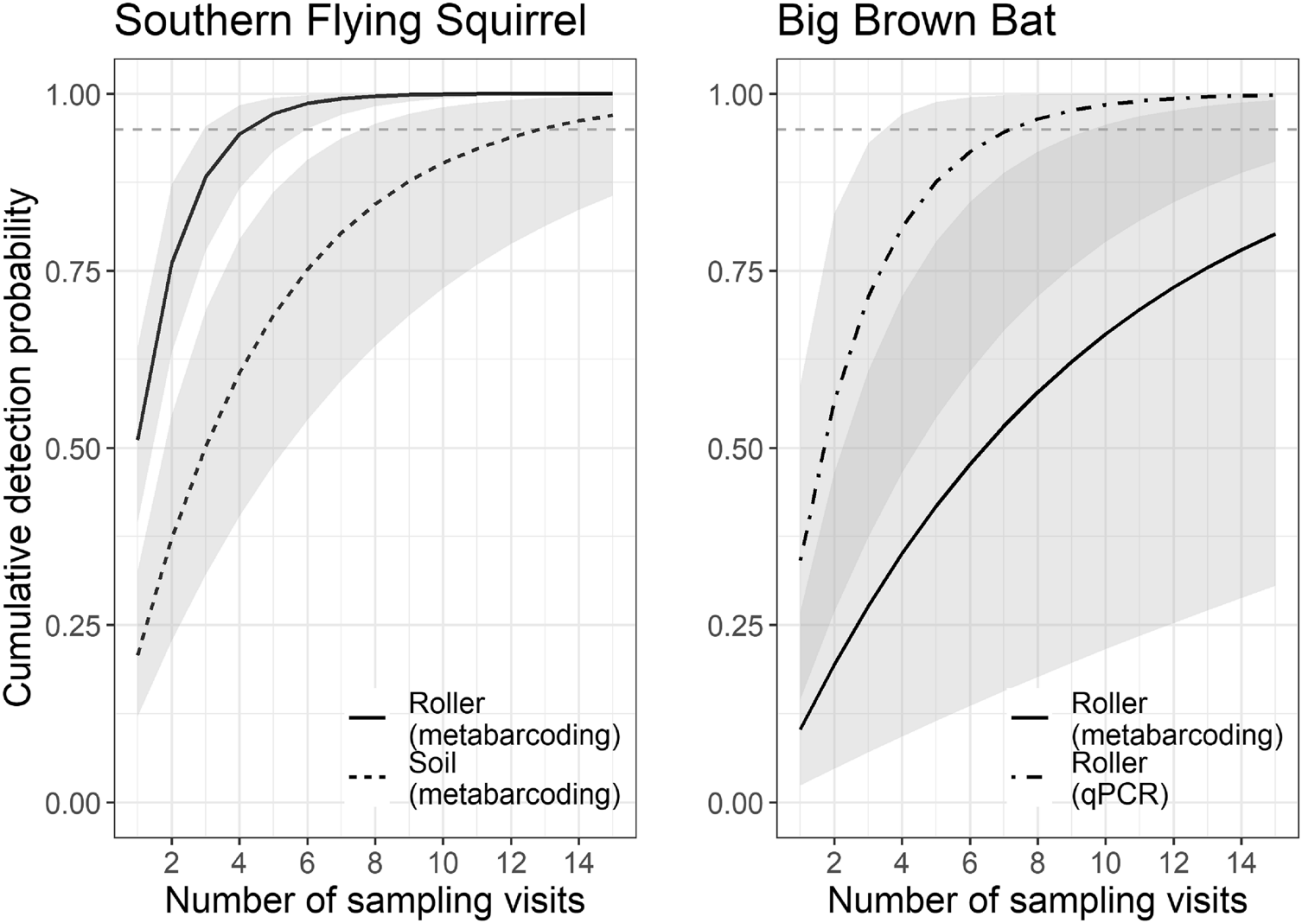
The cumulative probability of detecting a southern flying squirrel (left plot) or big brown bat (right plot) at least once at an occupied tree given increasing numbers of sampling visits. The plot, for southern flying squirrel, a cryptic arboreal species, illustrates the differences between tree bark surface (‘roller’) versus soil eDNA collection methods within a metabarcoding detection framework. The plot for big brown bat illustrates the difference between qPCR and metabarcoding molecular detection methods on samples collected using roller eDNA techniques. Shaded areas show 95% credible intervals. The gray dashed horizontal line represents a cumulative 95% certainty of detecting the species at least once.

Arboreal mammal species with the highest detection probability according to the community occupancy model were also the species with the highest read counts (Figs. 1B and 4): gray squirrel (roller: 0.70; soil: 0.39), southern flying squirrel (roller: 0.51; soil: 0.21), and eastern chipmunk (roller: 0.50; soil; 0.23). Surprisingly, the non-arboreal white-tailed deer also showed relatively high detection probability estimates within both roller (0.58) and soil (0.23) samples (Fig. 4). Based on these estimates for southern flying squirrel, the most cryptic non-bat arboreal mammal that we detected, ~ 4 roller sampling visits to an ‘occupied’ tree would be needed to ensure 95% confidence of detection, compared with ~ 12 visits required with our soil eDNA methods (Fig. 5). The reduced data set included in our parallel analysis contained two fewer species (lacked American red squirrel and eastern cottontail). Modeling based on this data set yielded very similar results to those of the full data set (see Supplemental Figs. S2–4).

For big brown bat, metabarcoding detections occurred in 3 of 94 samples (all roller samples) at 3 of 21 trees, while qPCR detections occurred in 10 samples (9 roller samples and 1 soil sample) at 6 trees. Together, metabarcoding and qPCR detected big brown bat DNA in 11 samples at 7 trees, including one of the two trees identified as likely containing a roosting individual of this species. The multimethod occupancy model revealed that detection probability was 3.4 × higher using qPCR-based detection methods (49%, 95% CI = [21%, 86%]) than when using metabarcoding methods (15%, [3%, 41%]). Based on these estimates, ~ 7 visits to an occupied tree would be required to establish presence of big brown bats with 95% confidence using qPCR methods, while ~ 27 visits would be required using metabarcoding methods (Fig. 5).

## Discussion

Fully terrestrial eDNA sampling approaches offer a potentially powerful addition to biodiversity monitoring efforts^23,24^. However, protocols for using eDNA-based methods to characterize terrestrial biodiversity, and vertebrate communities in particular, are still nascent^27,28,30^. In this study, we show for the first time that an eDNA metabarcoding approach can be used to broadly characterize tree-dwelling mammal communities by sampling tree trunks and surrounding soil. Our findings add to recent work (e.g., for reptiles^22,24^) showing that surface eDNA collection methods, which are relatively untested compared with soil-based eDNA methods, can also be effective at detecting terrestrial vertebrates. Further, we demonstrate that supplementing metabarcoding detection with qPCR-based methods can greatly improve sensitivity, a potentially important consideration for monitoring schemes focused on rare taxa (e.g., Refs.^11,12^). Together, our results have significant implications for global biodiversity conservation as the broader guild of arboreal vertebrates includes highly threatened^5,48,49^, as well as invasive alien species^50^, that are often cryptic, inhabit inaccessible locations, and are therefore challenging to monitor.

Our methods captured over 60% of the mammalian diversity expected at the sites, and a similar fraction of the subset of arboreal species, despite sampling only 21 trees. Species accumulation curves suggest that more species would likely have been added with increased sampling effort. These results broadly agree with those of Leempoel et al.^23^ who found that soil eDNA metabarcoding well characterized mammal communities in California chaparral. However, in both our study and that of Leempoel et al.^23^, some conspicuous absences were evident. Bats comprised all of the arboreal species that we expected but failed to detect at our sites using metabarcoding (Fig. 1A). Leempoel et al.^23^ also noted a lack of bat detections (2 of 14 possible taxa detected), which they suggested could be due to low efficiency of either the 12S primer set or of their soil sampling methods for that order. While both reasons could also apply to the lack of bats detected in our study (discussed further below), the performance of the 12S primer set very likely contributed to our lack of American black bear detections as MiMammal-U primers are known to be ineffective at amplifying bear DNA^38^. These challenges highlight the reality that false negatives and varying detectability among species are common issues to all survey approaches, including eDNA metabarcoding. Our study represents a rare example among metabarcoding studies in that it uses repeated sampling and community occupancy models to quantify false negative rates. This quantitative approach, coupled with continued experimentation with different molecular techniques and survey methods (e.g., Refs.^23,27^), will be vital to helping researchers decide how eDNA metabarcoding methods will fit into existing biodiversity monitoring efforts moving forward.

Although our results suggest that sampling for tree-roosting bats using eDNA metabarcoding still requires further research and optimization, our approach likely has application to characterizing communities in a much broader range of arboreal species globally. Geographic regions with multiple elusive arboreal mammals of management interest—for example, gliders and tree kangaroos in Australasia, or primates in the global tropics—may be particularly suited for a metabarcoding approach for community-level assessments^4,8,9,49^. It may be especially useful for rapid biodiversity assessments (RBAs^51^) in remote forested environments, where the ability to collect multiple samples relatively rapidly without regard to time of day would be a key advantage^27^. Existing survey methods to monitor arboreal mammals tend to be optimized for particular groups of species, often segregated by body size and behavior, with no suitable single method available to characterize all members of the guild^4,8,9,16,49^. Diurnal and nocturnal species, for example, often require separate survey methods or timing^8^. While camera traps capture both diurnal and nocturnal species, they typically miss smaller species^16,23^. The need for multiple methods to survey for nocturnal and diurnal, or large and small, species separately raises the cost of sampling and can result in datasets that are difficult to compare across sites because of inherent sampling biases^8^. Excluding bats, we found encouraging results for both diurnal and nocturnal arboreal species of a broad range of body sizes, detecting all seven expected species (Fig. 1A). While more work is needed to assemble robust genetic reference libraries before global arboreal mammal monitoring with eDNA metabarcoding will be broadly feasible, a clear advantage of the technique remains the power to detect a broad swath of species, with widely varying morphologies and behaviors, with a single method^23,27,28,51^.

The promise of eDNA metabarcoding approaches for at least some arboreal guilds is well illustrated by our results for southern flying squirrel, *Glaucomys volans*. Like other flying squirrels (Tribe: Pteromyini), this species is strictly nocturnal, highly arboreal, and tends to get injured in live traps, making it difficult to directly observe and monitor^48,52^. Yet *G. volans* eDNA was readily detectable using metabarcoding in our study, occurring in 19–26% of soil samples and 47–52% of roller samples across both sites. Our similarly encouraging results for detecting other squirrels (Sciuridae) also bode well for management applications. For example, the methods would enable fine-scale mapping of habitat use in places such as the United Kingdom where native red squirrels (*Sciurus vulgaris*) are outcompeted by eastern gray squirrels, or the Delmarva peninsula (USA) to support the conservation efforts for the Delmarva fox squirrel (*Sciurus niger cinereus*)^53^. Further research is needed to determine the extent to which our results for squirrels generalize to other taxa with similar active tree-climbing lifestyles (e.g., gliders^4^, primates^49^).

Our finding that soil samples revealed fewer species, had lower detection probability, and had lower read counts than roller samples, even for some non-arboreal species like white-tailed deer, likely reflects multiple factors. First, soil and tree bark represent markedly different biological and chemical environments that likely differ in eDNA quantity by species, eDNA persistence rates^54,55^, and microorganism abundance. The latter may be especially pertinent to our study as we observed a relatively large drop in the number of reads after removal of microorganism reads, especially for soil samples. This suggests that performing additional purification steps prior to sequencing could boost the ability of both methods, and especially soil eDNA, to detect target species by increasing mammalian sequencing depth. Other in-lab factors, such as method of extraction^23^ or choice of primers, similarly have the potential to influence the recovery and amplification of target species’ DNA and should be the focus of future research.

Next, our focal trees were not chosen to occur near any special attractants or areas of multi-species use, such as saltlicks or water sources, which has proven successful in other vertebrate eDNA studies^18,25,31,32,56^. It is possible that adding a broader range of soil sampling sites, including some targeted towards other guilds (e.g., burrow users^32,56^), would have yielded a more complete inventory. Nevertheless, both soil and surface methods have advantages over the much more commonly-used metabarcoding approaches that rely on natural water bodies for assessing mammal communities^16,17,27–30,38^ as they are not limited to where these features occur. Our study is the first to suggest that surface eDNA metabarcoding methods can be a powerful supplement to established soil-based methods of characterizing mammal communities, especially for arboreal species.

As noted, bats were especially lacking from our eDNA metabarcoding results, with only two of six likely species detected. Notably, our metabarcoding species list lacked two of the bat species that our sampling scheme was designed around (eastern red bat and northern long-eared bat) and for which we had confirmed recent presence at the sites (Table 1). The lack of northern long-eared bat detections may directly relate to recent precipitous population declines (~ 99%) caused by white-nose syndrome^57^. However, the lack of eastern red bat detections was especially surprising as roosting of this species was suspected based on telemetry in 17 of our 21 target trees. Reasons for this omission may relate to the fact that eastern red bats roost singly on small twigs and in leaf clusters, and therefore may not leave much DNA on tree trunks. Another possibility is low efficiency of the 12S primer set for bats, although we were unable to find information about this in the literature. It is notable that Leempoel et al.^23^ had a similarly poor representation of bats with comparable soil-based methods. However, our metabarcoding results did indicate that we are capable of detecting even uncommon, or at least unexpected, bat species with our methods. Eastern small-footed bat, which is typically viewed as a rock-roosting species and is considered endangered by the International Union for Conservation of Nature (IUCN)^58^, was detected in both soil and surface eDNA samples from Morristown National Historic Park. This species was not otherwise confirmed as present at the site until a year later, in spring 2022, when it was caught in a mist net (BM, unpublished data). Our results with respect to bat detections, along with those of others^23^, underscore the need for further research to adapt eDNA metabarcoding methods to this vulnerable group, which could contribute much needed demographic and distribution information. This is especially urgent as 18% of bat species are listed as “data deficient” by the IUCN, while 57% lack basic population trend information^5,58^.

Our comparison of qPCR to metabarcoding detection methods for big brown bat represents a hopeful result for the use of eDNA to monitor rare vertebrates that are of particular conservation interest. It is well-known that qPCR-based eDNA surveys targeted towards individual species return higher detection probabilities and have greater power at low abundance, than metabarcoding approaches^59^. Our results agree, showing for the first time that adding a qPCR step in the analysis of surface and soil eDNA samples can be effective for detecting bats in forested environments. The addition of a qPCR step opens the door for developing species-specific assays to increase detection power for endangered or elusive bat species, or other cryptic arboreal mammals^49^. Emerging molecular detection approaches such as droplet digital PCR have the potential to increase this sensitivity even further^59^. Like other eDNA-based tools and survey tools in general, careful consideration of sampling effort, the natural history of target species, and the configuration of different field and molecular methods will be key to optimizing our approach to characterize mammal communities, or to target a particular species, in different regions.

Although eDNA surveys are not inexpensive given the need for both fieldwork and molecular analyses, they can be cheaper than conventional approaches, especially if such approaches require many hours of fieldwork or expensive equipment^60^. Thus, the relative cost-effectiveness of surface or soil eDNA surveys will depend heavily on the mammal communities of interest, the mix of methods that must be employed to effectively sample them, and the purpose of the sampling efforts. However, even if costs are increased, eDNA surveys can reduce field time to the extent that they can improve detection rates, either by replacing or supplementing conventional sampling methods (e.g., as a supplement to visual observations). With higher detection rates, fewer visits are required to achieve the same results. This operational efficiency would be especially advantageous when field conditions present safety risks, are intrusive to sensitive habitats, or are challenging to access. For example, adding surface eDNA sampling to existing visual surveys of eastern wood rats (*Neotoma floridana*), a cryptic mammal that inhabits steep, rocky slopes in the eastern US, could likely increase detection power, thereby reducing the need for additional risky and costly sampling visits. More studies involving direct comparisons among methods (e.g., Refs.^23,24,30,60^), in a variety of ecoregions, are needed to determine the extent to which incorporating our methods into existing vertebrate monitoring workflows would increase efficiency.

Finally, we detected other vertebrates, including seven birds and one salamander, in soil and surface eDNA samples, despite our use of a mammal-specific primer set. This is similar to results from Leempoel et al.^23^ in California using the same primer set, in which six bird species were detected. We found that surface eDNA detected more bird species than soil, perhaps for the same reasons as for mammals (above). Our results provide evidence that surface eDNA surveys, with taxon-specific primers, could be used to survey bird communities, or used to target particularly rare species in forested ecosystems (e.g., Ref.^61^). Our detection of a salamander, coupled with recent promising research into reptile detection using surface eDNA methods^22,24^ suggests a broader potential for applications with other vertebrates as well. Finally, both surface and soil eDNA metabarcoding can be expanded beyond forests, providing insight into their effectiveness in other habitats (e.g., caves^17^ or talus slopes). Our study and others highlight that the potential of coupling surface and soil eDNA methods for detecting and monitoring mammalian biodiversity, and terrestrial organisms generally, has yet to be fully realized.

## Supplementary Information

Supplementary Information 1.

Supplementary Information 2.

## Data Availability

Raw sequence data for this project are available at NCBI BioProject PRJNA915402: https://www.ncbi.nlm.nih.gov/bioproject/PRJNA915402. Processed data are available on Open Science Foundation: https://doi.org/10.17605/OSF.IO/A6FYV.

## Code availability

R code and data to reproduce the results and figures in this paper are available on GitHub (https://github.com/mikeallen-eco/mammal/) with an archived copy on Zenodo (https://doi.org/10.5281/zenodo.7486742).

## References

[1] Ceballos G, Ehrlich PR, Soberón J, Salazar I, Fay JP (2005). Global mammal conservation: What must we manage?. Science.

[2] Carwardine J (2008). Cost-effective priorities for global mammal conservation. Proc. Natl. Acad. Sci..

[3] Visconti P (2011). Future hotspots of terrestrial mammal loss. Philos. Trans. R. Soc. B Biol. Sci..

[4] Cripps JK (2021). Double-observer distance sampling improves the accuracy of density estimates for a threatened arboreal mammal. Wildl. Res..

[5] Frick WF, Kingston T, Flanders J (2020). A review of the major threats and challenges to global bat conservation. Ann. N. Y. Acad. Sci..

[6] Weller TJ, Cryan PM, O’Shea TJ (2009). Broadening the focus of bat conservation and research in the USA for the 21st century. Endanger. Species Res..

[7] Holland GJ (2012). Conservation cornerstones: Capitalising on the endeavours of long-term monitoring projects. Biol. Conserv..

[8] Bowler MT, Tobler MW, Endress BA, Gilmore MP, Anderson MJ (2017). Estimating mammalian species richness and occupancy in tropical forest canopies with arboreal camera traps. Remote Sens. Ecol. Conserv..

[9] Pocknee CA, Lahoz-Monfort JJ, Martin RW, Wintle BA (2021). Cost-effectiveness of thermal imaging for monitoring a cryptic arboreal mammal. Wildl. Res..

[10] Dambly LI, Jones KE, Boughey KL, Isaac NJ (2021). Observer retention, site selection and population dynamics interact to bias abundance trends in bats. J. Appl. Ecol..

[11] de Torrez ECB, Ober HK, McCleery RA (2016). Use of a multi-tactic approach to locate an endangered Florida bonneted bat roost. Southeast. Nat..

[12] Murray SW, Kurta A (2004). Nocturnal activity of the endangered Indiana bat (*Myotis sodalis*). J. Zool..

[13] Whisson DA, McKinnon F, Lefoe M, Rendall AR (2021). Passive acoustic monitoring for detecting the Yellow-bellied Glider, a highly vocal arboreal marsupial. PLoS ONE.

[14] Gregory T, Carrasco Rueda F, Deichmann J, Kolowski J, Alonso A (2014). Arboreal camera trapping: Taking a proven method to new heights. Methods Ecol. Evol..

[15] Chambers CL, Vojta CD, Mering ED, Davenport B (2015). Efficacy of scent-detection dogs for locating bat roosts in trees and snags. Wildl. Soc. Bull..

[16] Mena JL (2021). Environmental DNA metabarcoding as a useful tool for evaluating terrestrial mammal diversity in tropical forests. Ecol. Appl..

[17] Serrao NR, Weckworth JK, McKelvey KS, Dysthe JC, Schwartz MK (2021). Molecular genetic analysis of air, water, and soil to detect big brown bats in North America. Biol. Conserv..

[18] Newton JP, Bateman PW, Heydenrych MJ, Mousavi-Derazmahalleh M, Nevill P (2022). Home is where the hollow is: Revealing vertebrate tree hollow user biodiversity with eDNA metabarcoding. Environ. DNA.

[19] Luszcz TM (2016). A blind-test comparison of the reliability of using external morphology and echolocation-call structure to differentiate between the little brown bat (*Myotis lucifugus*) and Yuma myotis (*Myotis yumanensis*). Northwest. Nat..

[20] Rees HC, Maddison BC, Middleditch DJ, Patmore JR, Gough KC (2014). The detection of aquatic animal species using environmental DNA–a review of eDNA as a survey tool in ecology. J. Appl. Ecol..

[21] Padgett-Stewart TM (2016). An eDNA assay for river otter detection: A tool for surveying a semi-aquatic mammal. Conserv. Genet. Resour..

[22] Matthias L, Allison MJ, Maslovat CY, Hobbs J, Helbing CC (2021). Improving ecological surveys for the detection of cryptic, fossorial snakes using eDNA on and under artificial cover objects. Ecol. Indic..

[23] Leempoel K, Hebert T, Hadly EA (2020). A comparison of eDNA to camera trapping for assessment of terrestrial mammal diversity. Proc. R. Soc. B.

[24] Kyle KE (2022). Combining surface and soil environmental DNA with artificial cover objects to improve terrestrial reptile survey detection. Conserv. Biol..

[25] Kinoshita G, Yonezawa S, Murakami S, Isagi Y (2019). Environmental DNA collected from snow tracks is useful for identification of mammalian species. Zool. Sci..

[26] Marucco F, Boitani L, Pletscher DH, Schwartz MK (2011). Bridging the gaps between non-invasive genetic sampling and population parameter estimation. Eur. J. Wildl. Res..

[27] Coutant O (2021). Amazonian mammal monitoring using aquatic environmental DNA. Mol. Ecol. Resour..

[28] Sales NG (2020). Fishing for mammals: Landscape-level monitoring of terrestrial and semi-aquatic communities using eDNA from riverine systems. J. Appl. Ecol..

[29] Harper LR (2019). Environmental DNA (eDNA) metabarcoding of pond water as a tool to survey conservation and management priority mammals. Biol. Conserv..

[30] Lyet A (2021). eDNA sampled from stream networks correlates with camera trap detection rates of terrestrial mammals. Sci. Rep..

[31] Ishige T (2017). Tropical-forest mammals as detected by environmental DNA at natural saltlicks in Borneo. Biol. Conserv..

[32] Ryan E, Bateman P, Fernandes K, van der Heyde M, Nevill P (2022). eDNA metabarcoding of log hollow sediments and soils highlights the importance of substrate type, frequency of sampling and animal size, for vertebrate species detection. Environ. DNA.

[33] Valentin RE (2020). Moving eDNA surveys onto land: Strategies for active eDNA aggregation to detect invasive forest insects. Mol. Ecol. Resour..

[34] Collins BR, Anderson K (1994). Plant Communities of New Jersey: A Study in Landscape Diversity.

[35] Cove, M. V. *et al.* SNAPSHOT USA 2019: A coordinated national camera trap survey of the United States (2021). 10.1002/ecy.3353 33793977

[36] iNaturalist. iNaturalist Research-grade Observations. https://inaturalist.org/. Occurrence dataset 10.15468/ab3s5x. Accessed via https://gbif.org/. 14 June 2022 (2022).

[37] Illumina I (2013). 16S Metagenomic sequencing library preparation. Prep. 16S Ribosomal RNA Gene Amplicons Illumina MiSeq Syst..

[38] Ushio M (2017). Environmental DNA enables detection of terrestrial mammals from forest pond water. Mol. Ecol. Resour..

[39] Boyer F (2016). obitools: A unix-inspired software package for DNA metabarcoding. Mol. Ecol. Resour..

[40] Cunningham F (2022). Ensembl 2022. Nucleic Acids Res..

[41] Johnson M (2008). NCBI BLAST: A better web interface. Nucleic Acids Res..

[42] Kéry M, Royle JA (2016). Applied Hierarchical Modeling in Ecology: Analysis of Distribution, Abundance and Species Richness in R and BUGS: Volume 1: Prelude and Static Models.

[43] Foster ZS, Sharpton TJ, Grünwald NJ (2017). Metacoder: An R package for visualization and manipulation of community taxonomic diversity data. PLoS Comput. Biol..

[44] Oksanen, J. *et al.* ‘vegan’: Community ecology package. R package version 2.5-7. https://CRAN.R-project.org/package=vegan (2020).

[45] Nichols JD (2008). Multi-scale occupancy estimation and modelling using multiple detection methods. J. Appl. Ecol..

[46] Kellner, K. Package ‘jagsUI’: A wrapper around ‘rjags’ to Streamline ‘JAGS’ analyses. R Package Version 1.5.1. (2019).

[47] R Core Team. R: A language and environment for statistical computing. Vienna, Austria: R Foundation for Statistical Computing. https://www.R-project.org/ (2021).

[48] Clucas B, Atkins Z (2022). Using camera traps to survey Humboldt’s flying squirrels in old- and second-growth redwood forests. Northwest. Nat..

[49] Oliver K, Ngoprasert D, Savini T (2020). Assessment of survey protocol for estimates of abundance for elusive nocturnal primates. Wildl. Res..

[50] Boback SM, Nafus MG, Yackel Adams AA, Reed RN (2020). Use of visual surveys and radiotelemetry reveals sources of detection bias for a cryptic snake at low densities. Ecosphere.

[51] Yu DW (2012). Biodiversity soup: Metabarcoding of arthropods for rapid biodiversity assessment and biomonitoring. Methods Ecol. Evol..

[52] Suzuki KK, Ando M (2019). Early and efficient detection of an endangered flying squirrel by arboreal camera trapping. Mammalia.

[53] Greene DU, McCleery RA, Wagner LM, Garrison EP (2016). A comparison of four survey methods for detecting fox squirrels in the southeastern United States. J. Fish Wildl. Manag..

[54] Valentin RE, Kyle KE, Allen MC, Welbourne DJ, Lockwood JL (2021). The state, transport, and fate of aboveground terrestrial arthropod eDNA. Environ. DNA.

[55] Barnes MA, Turner CR (2016). The ecology of environmental DNA and implications for conservation genetics. Conserv. Genet..

[56] Katz AD (2021). Environmental DNA is effective in detecting the federally threatened Louisiana Pinesnake (*Pituophis ruthveni*). Environ. DNA.

[57] Hoyt JR, Kilpatrick AM, Langwig KE (2021). Ecology and impacts of white-nose syndrome on bats. Nat. Rev. Microbiol..

[58] Wieringa JG (2022). Comparing predictions of IUCN Red List categories from machine learning and other methods for bats. J. Mammal..

[59] Wood SA (2019). A comparison of droplet digital polymerase chain reaction (PCR), quantitative PCR and metabarcoding for species-specific detection in environmental DNA. Mol. Ecol. Resour..

[60] Smart AS (2016). Assessing the cost-efficiency of environmental DNA sampling. Methods Ecol. Evol..

[61] Day K (2019). Development and validation of an environmental DNA test for the endangered Gouldian finch. Endanger. Species Res..

