# Supplementary material for: Sampling environmental DNA from trees and soil to detect cryptic arboreal mammals
Source: Sci Rep. 2023 Jan 5;13:180. doi: 10.1038/s41598-023-27512-8 (PMC9814459; doi:10.1038/s41598-023-27512-8)
Supplement: Supplementary file 2 — Supplementary Information 2. [file 41598_2023_27512_MOESM2_ESM.pdf]

Sampling environmental DNA from trees and soil to detect cryptic arboreal mammals (Supplementary Material)

Michael C. Allen[1*], Robert Kwait[1], Anthony Vastano[1], Alex Kisurin[1], Isabelle Zoccolo[1], Benjamin D. Jaffe[2], Jordan C. Angle[3], Brooke Maslo[1], and Julie L. Lockwood[1]

[1]Ecology, Evolution and Natural Resources, Rutgers University, 14 College Farm Road, New Brunswick, NJ 08902
[2]ExxonMobil Biomedical Sciences Inc, Annandale, New Jersey USA
[3]ExxonMobil Upstream Research Company, Spring, Texas USA
[*]Corresponding Author: michael.allen@rutgers.edu

Appendix S1. Assembling local DNA reference library for bat species

To assemble a supplemental DNA reference library for local bat species, we took 2-3 mm wing punch biopsies from at least one member of each of the 9 bat species held in collections at Rutgers University (see Supplemental Table S3). We extracted DNA from each wing punch using the DNeasy Blood and Tissue kit (Qiagen) following the manufacturers protocols. We ran PCRs using MiMammal-U (Ushio et al., 2017), then ran the resulting products on a slab gel to demonstrate the ability of the primer sets to amplify each species. Then we sent each sample for Sanger sequencing to determine whether or not either of these primers could distinguish between all the bat species. The MiMammal-U primer set targets an ~200 bp region of the 12S locus and was created to specifically amplify mammal DNA from environmental samples (Ushio et al., 2017).

For MiMammal-U, the reaction consisted of 12.5 µl of KAPA HiFi HotStart ReadyMix (Roche Molecular Systems, Inc., Branchburg, New Jersey, USA), 5 µl of each primer (0.1 µM), and 2.5 µl of DNA extract. The PCR cycling parameters were as follows: 98 °C for 3 min, 35 cycles of 95 °C (30 s), 65 °C (30 s), and 72 °C (1 min), followed by final extension at 72 °C for 5 min, and hold at 4°C. Following amplification and gel verification, we enzymatically cleaned the PCR product with ExoSAP-IT diluted 2:1 with PCR-grade water. Then we added 2 µl of the ExoSAP working solution to 10 µl of PCR product and ran the following protocol: 37 °C for 15 min, 80 °C for 15 min, and hold at 4 °C. We quantified the purified PCR products using the Qubit dsDNA high sensitivity DNA quantification assay (Invitrogen).

We prepared samples for Sanger sequencing by mixing approximately 10-12 ng of DNA with 2.5 µl of primer and the amount of PCR-grade water required to bring the reaction volume to 15 µl. A separate reaction was created for the forward and reverse of each primer. The resulting mixtures were sent to GENEWIZ (South Plainfield, New Jersey, USA) for Sanger sequencing.

We removed low quality bases from sequences returned by GENEWIZ, aligned forward and reverse reads, and generated consensus sequences using Geneious Prime software (Biomatters, Inc., Aukland, New Zealand). We then compared the resulting sequence against those in online databases using BLAST (Johnson et al., 2008). We also downloaded sequences from each of the 9 bat species that had available sequences from GenBank (see Supplemental Table S3). We generated consensus sequences for each species from all individuals from our generated library and online sources to mark polymorphisms. Then we aligned all full species consensus

sequences to look for differences among species. We considered species distinguishable if they had at least 5 non-ambiguous differences in sequence.

Literature Cited

Johnson, M., Zaretskaya, I., Raytselis, Y., Merezhuk, Y., McGinnis, S., & Madden, T. L. (2008). NCBI BLAST: a better web interface. Nucleic Acids Research, 36(suppl_2), W5-W9.

Ushio, M., Fukuda, H., Inoue, T., Makoto, K., Kishida, O., Sato, K., Murata, K., Nikaido, M., Sado, T., Sato, Y., Takeshita, M., Iwasaki, W., Yamanaka, H., Kondoh, M., & Miya, M. (2017). Environmental DNA enables detection of terrestrial mammals from forest pond water. Molecular Ecology Resources, 17(6), e63-e75.

Table S1. Field sanitation procedures employed to reduce the likelihood of sample contamination.

Equipment / surface	When sanitation measure was performed	Description of sanitation measure performed
Paint rollers[a] & stainless steel spoons (for soil collection)	In the lab before going into the field	Cleaned off debris using tap water, soaked in 10% bleach for ≥ 10 min, rinsed under tap water for ≥ 30 s, and triple rinsed all with deionized (DI) water
Paint roller pole and paint roller frame (portion of pole where roller attaches)	In the lab before going into the field	Wiped with paper towel soaked in 10% bleach solution, followed by a DI water rinse
Paint rollers, stainless steel spoons, and paint roller frame	In the field, between samples	Transported within sterile plastic bags (Ward's, West Henrietta, New York, USA; VWR, Radnor, Pennsylvania, USA) to prevent contamination
Paint roller frame	In the field, between samples	Flame sterilized twice by spraying with 100% non-denatured ethanol and igniting; affixed a clean roller to the pole using a sterile bag and gloved hand. A sterile bag and gloved hand were also used to remove the roller.
Forceps	In the field, between samples	Flame-sterilized 2x as above.

[a]152 mm (6 inch) long 'mini' paint rollers with 6 mm (0.25 inch) woven synthetic nap (Linzer Products Corporation, West Babylon, New York, USA).

Table S2. Sampling information for the 21 trees sampled during a 2021 eDNA metabarcoding study of mammals in Rutgers Ecological Preserve ('Rutgers') and Morristown National Historic Park ('Morristown') in New Jersey, USA.

					Sample summary for each visit[b]		
**Focal tree**	**Site**	**Tree species**	**Target bat species[a]**	**Date of 1st sample (2021)**	**1**	**2**	**3**
1	Rutgers	*Quercus coccinea*	Eastern red	8 July	Roller / Soil	Roller / Soil	Roller / Soil
2	Rutgers	*Ulmus americana*	Eastern red	9 July	Roller	Roller	Roller
3	Rutgers	*Acer saccharum*	Eastern red	10 July	Roller	Roller	Roller
4	Rutgers	*Betula lenta*	Eastern red	11 July	Roller	Roller	Roller
5	Morristown	*Ailanthus altissima*	Eastern red	15 July	Roller	Roller	Roller
6	Morristown	Unknown	Eastern red	16 July	Roller	Roller	Roller
7	Morristown	*Quercus alba*	Eastern red	6 August	Roller	Roller	Roller
8	Morristown	*Liriodendron tulipifera*	Eastern red	6 August	Roller	Roller / Soil	Roller
9	Morristown	*Quercus alba*	Big brown	6 August	Roller / Soil	Roller / Soil	Roller / Soil
10	Morristown	*Fagus grandifolia*	Big brown	6 August	Roller / Soil	Roller / Soil	Roller / Soil
11	Morristown	*Liriodendron tulipifera*	Eastern red	7 August	Roller / Soil	Roller / Soil	Roller / Soil
12	Morristown	*Liriodendron tulipifera*	Eastern red	7 August	Roller / Soil	Roller / Soil	Roller
13	Morristown	*Liriodendron tulipifera*	Eastern red	8 August	Roller / Soil	Roller / Soil	Roller / Soil
14	Morristown	*Fagus grandifolia*	Eastern red	8 August	Roller / Soil	Roller / Soil	Roller / Soil
15	Morristown	*Liriodendron tulipifera*	Eastern red	9 August	Roller	No sample	No sample
16	Morristown	*Betula lenta*	Eastern red	9 August	Roller / Soil	No sample	No sample
17	Rutgers	*Fagus grandifolia*	Eastern red	11 August	Roller / Soil	Roller / Soil	Roller / Soil
18	Rutgers	Unknown	Eastern red	12 August	Roller	Roller	Roller / Soil
19	Rutgers	Unknown	Eastern red	14 August	Roller / Soil	Roller / Soil	Roller / Soil

20	Rutgers	Unknown	N. long-eared	13 September	Roller / Soil	Roller / Soil	Roller / Soil
21	Rutgers	*Quercus rubra*	N. long-eared	13 September	Roller / Soil	Roller / Soil	Roller / Soil

[a]Bat species that was tracked with radio telemetry and was believed to be roosting in the tree during the 1st sampling visit. For scientific names, see Table S4.

[b]These columns indicate which sample types were collected, extracted, and analyzed at each tree during each of the three sampling visits (1 –taken within 24 hr of a bat being tracked to the tree; 2 –taken within 48 hr; 3 – taken within 72 hr). Roller / Soil – both roller and soil samples were collected, extracted, and analyzed; Roller – only roller samples extracted and analyzed; No sample – no soil or roller samples were collected.

Table S3. Summary of bioinformatics steps employed in this study, as adapted from OBITools documentation and Leempoel et al. (2020)[b].

Bioinformatics step	Description
1	Aligned forward and reverse reads using Illumina paired-end alignment
2	Removed all unpaired reads and reads with join scores lower than 40
3	Removed adapter sequences and bases with quality scores < 30 using CUTADAPT.
4	Removed reads with ≥ 21 ambiguous bases using PRINSEQ so that primer sequences could be differentiated from strings of ambiguous bases
5	Used *ngsfilter* and *obiuniq* within OBITools to assign reads to sample and de-replicate them, removing all unassigned reads
6	Removed all sequences having a read count < 10 or that were shorter than 80 bp in length and filtered all reads for PCR or sequencing errors
7	Generated a reference database from all vertebrate sequences available from the Ensembl database (www.ensembl.org)
8	Used EcoPCR to simulate a PCR on the database with the MiMammal-U primer set
9	Removed duplicates and sequences that did not have an assignment to at least family level from the resulting database

[a]Leempoel, K., Hebert, T., & Hadly, E. A. (2020). A comparison of eDNA to camera trapping for assessment of terrestrial mammal diversity. Proceedings of the Royal Society B, 287(1918), 20192353.

Table S4. List of bat species for which additional genetic material was acquired to be included in an eDNA metabarcoding survey for mammals in New Jersey, USA woodlands.

				Roost Type				
**Species**	**# tissue samples**	**Regions**	**No. sequences in GenBank**	**Foliage**	**Crevice**	**Bark**	**Rock**	**Roosting Behavior**
Big brown bat (*Eptesicus fuscus*)	1	NJ	4		X	X		Gregarious
Eastern Red bat (*Lasiurus borealis*)	1	NJ	3	X				Solitary
Northern Long-eared Bat (*Myotis septentrionalis*)	1	NJ	3		X	X		Gregarious
Little Brown Bat (*Myotis lucifugus*)	5	NJ, NY, VM, KY	0		X	X		Gregarious
Indiana Bat (*Myotis sodalis*)	5	NY, MI, AR, KY	0		X	X		Gregarious
Eastern small-footed bat (*Myotis leibii*)	1	KY	1				X	Solitary
Tricolored bat (*Perimyotis subflavus*)	1	KY	0	X	X	X		Gregarious
Hoary Bat (*Lasiurus cinereus*)	1	NJ	1	X				Solitary
Silver-haired bat (*Lasionycteris noctivagans*)	1	NJ	4	X		X		Solitary

Table S5. Species for which sequences were excluded as likely contaminants because they are the subject of active genetic research in Rutgers lab. Little brown bat is the only species from the list that would be expected to occur in terrestrial habitats in our study sites.

Group	Common Name	Scientific name
Fish	Blueback herring	*Alosa aestivalus*
	Tessellated darter	*Etheostoma olmstedii*
	Banded killifish	*Fundulus diaphanus*
	Largemouth bass	*Micropterus salmoides*
	Tadpole madtom	*Notorus gyrinus*
	Atlantic salmon	*Salmo salar*
Mammals	Indiana bat	*Myotis sodalis*
	Little brown bat	*Myotis lucifugus*
Reptiles	Little brown skink	*Scincella lateralis*

Table S6. Results of quality and contaminant removal filtering steps of mammal metabarcoding sequence data from samples collected in 2021 at Rutgers Ecological Preserve and Morristown National Historic Park, New Jersey, USA.[a]

	Roler eDNA samples		Soil eDNA samples		Negative control samples	
**Filter step**	**Total reads**	**Mean ± SE (n)**	**Total reads**	**Mean ± SE (n)**	**Total reads**	**Mean ± SE (n)**
Matched to index and with measurable read count	9,852,404	209,626 ± 11,541 (47)	4,731,187	135,177 ± 8,947 (35)	755,710	16,794 ± 2,776 (45)
Merging paired reads and initial quality filtering	6,281,995	133,660 ± 8,834 (47)	1,505,956	43,027 ± 3,006 (35)	569,152	12,648 ± 2,095 (45)
Removal of human, mouse, and bacterial reads	2,958,957	62,957 ± 7,276 (47)	34,381	1,433 ± 536 (24)	26,870	2,443 ± 936 (11)
Removal of other likely contaminant taxa	2,556,513	55,408 ± 7,116 (46)	33,387	1,452 ± 559 (23)	7073	1,415 ± 588 (5)
Subtraction of reads associated with negative control contamination and removal of OTUs with < 20 total reads across samples	2,548,768	55,408 ± 7,106 (46)	33,314	1,448 ± 558 (23)	0	0

[a]Sequences are from a total of 59 roller samples, 35 soil samples, and 46 negative control samples.

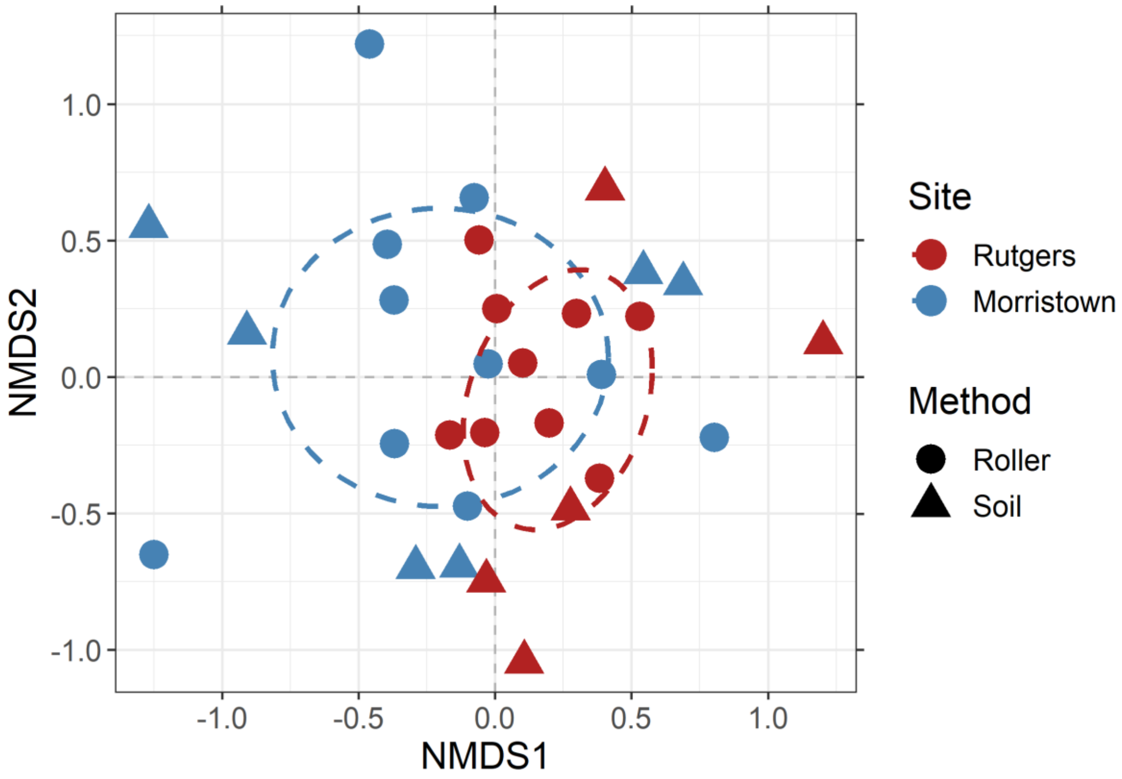

Figure S1. Non-metric multidimensional scaling ordination of mammal species abundances (based on metabarcoding read counts) at Rutgers Ecological Preserve and Morristown National Historic Park, New Jersey, USA. Circles represent sampling trees sampled using rollers, while triangles represent soil samples taken beneath trees. No clear separation is evident for roller versus soil samples, while the two study sites show moderate separation along the 1st NMDS axis (x-axis) as indicated by the 95% ellipses (dashed lines).

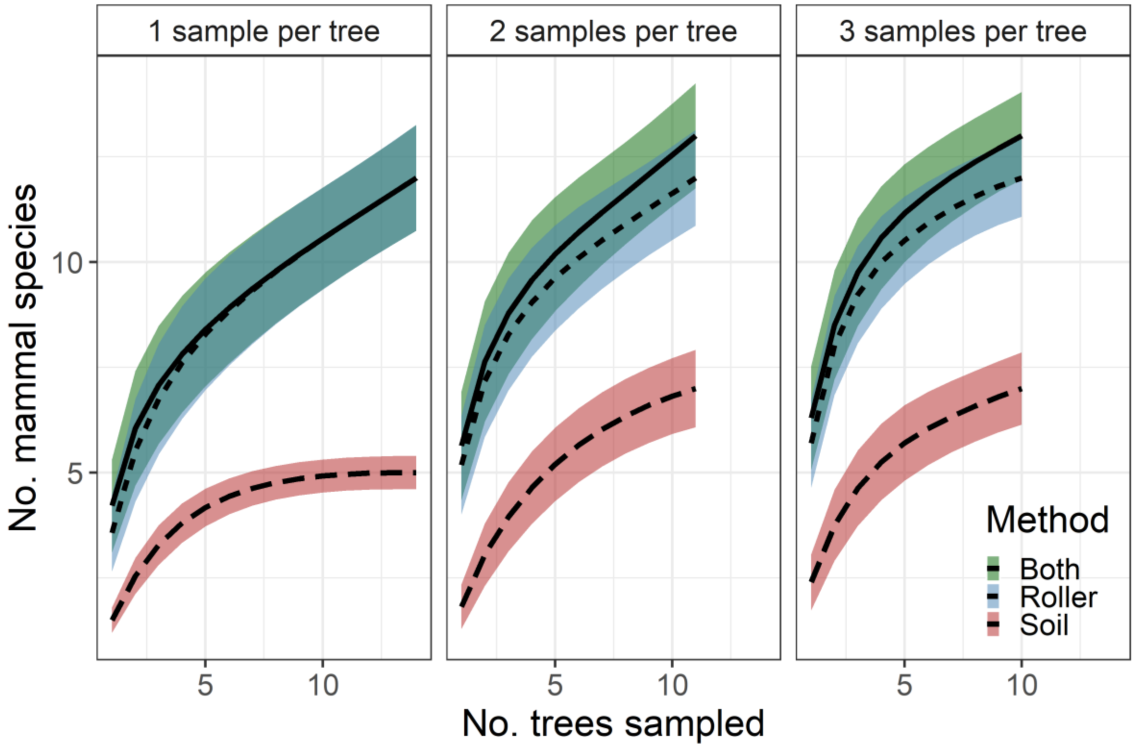

Figure S2. Species accumulation curves (mean ± 1 SD) for mammals detected using metabarcoding with tree bark surface ('roller') or soil eDNA sampling and varying numbers of sampling visits per tree (1-3). 'Both' refers to the pooled results from both roller and soil sampling. This figure shows the same model results as in Fig. 3 but based on a reduced data set that includes only the 35 samples (and 14 trees) with data from both soil and roller sampling.

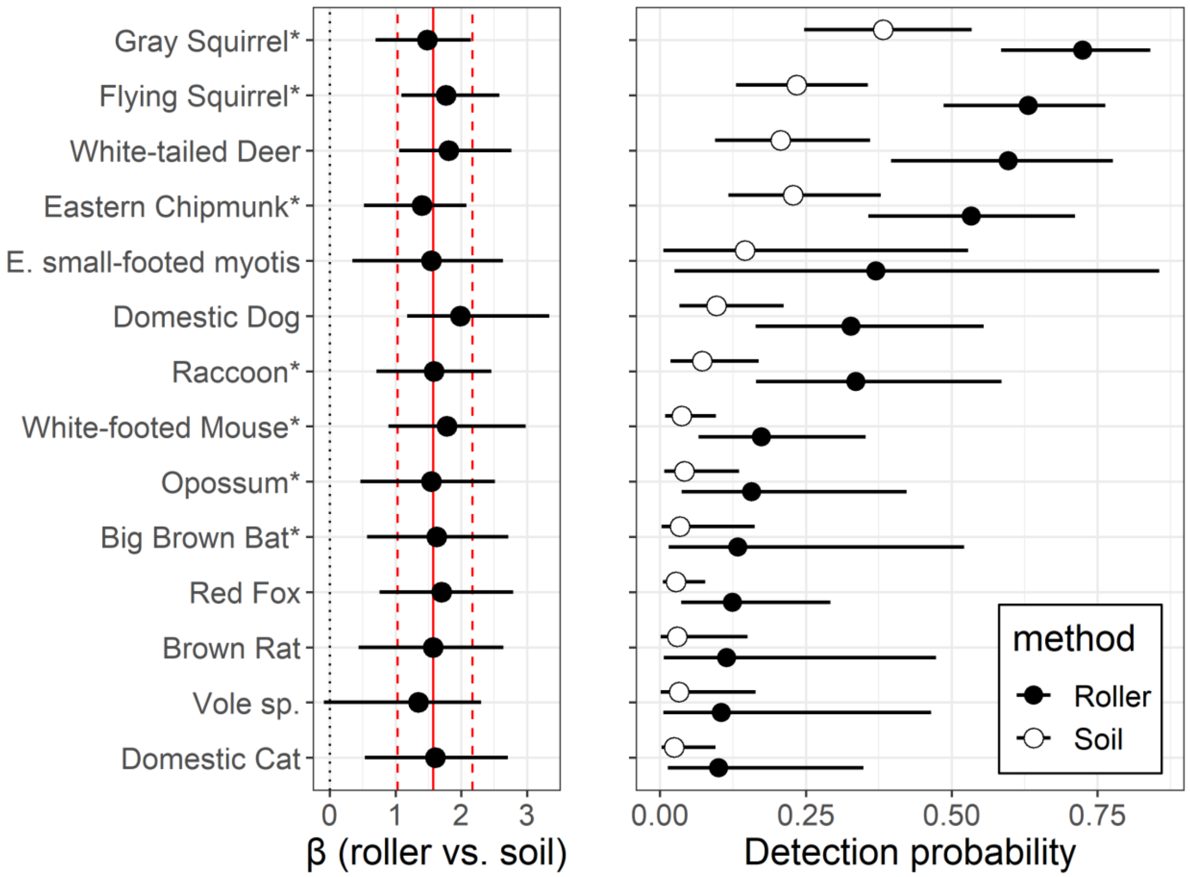

Figure S3. Results of a community occupancy model from a parallel analysis of mammal species detected via metabarcoding in New Jersey, USA woodlands. This figure shows the same model results as Fig. 4, but based on a reduced data set that includes only the 35 samples (and 14 trees) with both soil and tree bark surface ('roller') sampling data. Results were similar, though with wider credible intervals, to those from the model based on the full data set: β =1.57 (95% CI = [1.02, 2.17]) vs. 1.39 [0.85, 1.91]. As in Fig. 4, the left panel shows estimated slope coefficient for the effect of sampling method (roller vs. soil) on detection probability for each species (solid circles with 95% credible intervals) and for all species combined (solid vertical line with dashed lines indicating 95% credible intervals). A slope above zero (vertical dotted line) indicates higher detection probability for roller samples compared with soil samples. The right panel shows the model-estimated detection probability for each species with roller methods (solid circles) and soil (open circles) eDNA collection methods. Arboreal species are indicated with an asterisk (*).

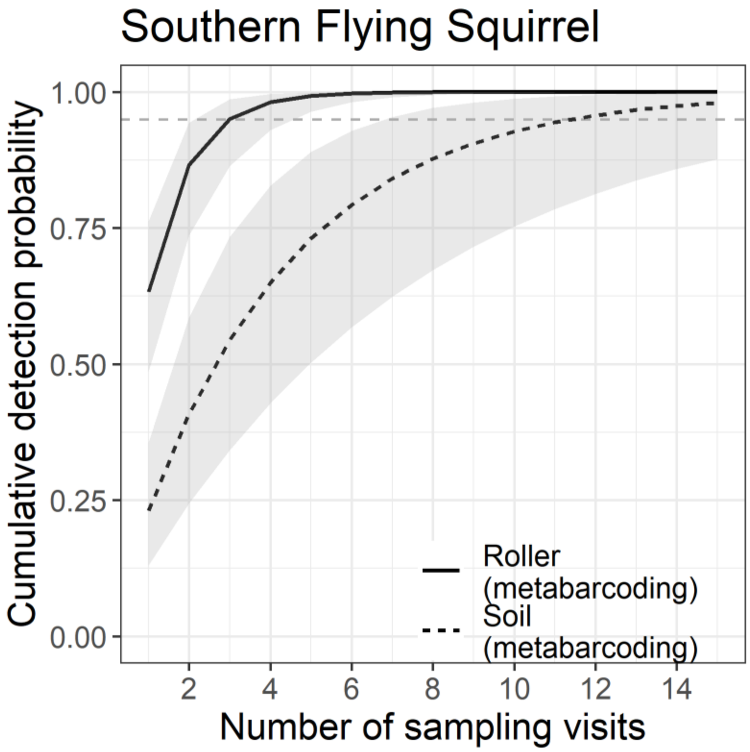

Figure S4. The cumulative probability of detecting a southern flying squirrel via eDNA metabarcoding at least once at an occupied tree given increasing numbers of sampling visits. This figure shows the same model results as in Fig. 5 but based on a reduced data set that includes only the 35 samples (and 14 trees) with data from both soil and tree bark surface ('roller') sampling. The plot illustrates that ~ 3 visits would be required with roller sampling, and ~ 11 with soil, to achieve 95% confidence in detecting southern flying squirrel at an occupied tree. This was generally similar to the results from the model based on the full data set (~ 4 and 12 visits, respectively; see Fig. 5). Shaded areas show 95% credible intervals. The gray dashed horizontal line represents a cumulative 95% certainty of detecting the species at least once.
